# Functional polymorphisms in cancer

**DOI:** 10.18632/oncoscience.129

**Published:** 2015-02-20

**Authors:** Divyansh Agarwal, Christos Hatzis, Lajos Pusztai

**Affiliations:** Department of Molecular, Cellular and Developmental Biology, Yale University, CT, U.S.A.

Germline variants (single nucleotide polymorphisms, SNPs) represent the vast majority of genetic changes occurring in cancer relative to the reference human genome. Two randomly chosen healthy individuals differ by approximately 20,000 germline variants in their exome [1]. In contrast, most solid tumors harbor an average of 30 to 100 somatic mutations that are considered potentially important in cancer biology. In breast cancer, the median number of non-synonymous mutations per cancer is 33 [1]. An unexpected observation from large scale sequencing efforts was the relative paucity of high frequency, recurrent somatic driver mutations in most solid tumors, which instead present a variable assortment of low frequency mutations in unique combinations in each cancer. These observations raise the possibility that inherited risk-modifying functional SNPs may interact with somatic mutations to collectively cause the malignant phenotype.

Inter-individual physiological and anatomical differences, to a large extent, are attributable to differences in germline variants, which illustrates the biological importance of functional polymorphisms. Disease-causing mutations are a subtype of SNPs that have profound deleterious effect on health, whereas other germline variants increase the risk for cancer development (e.g. BRCA1, CHEK2, FGF receptors, etc.) [2, 3]. The biological functional effect of the vast majority of human SNPs has not been studied yet. However, there is an increasing list of germ line variants that have been shown experimentally to impact protein function, without detectable association with disease states. For example, the M326I variant (rs3730089) in the p85α regulatory subunit of Phosphatidylinositol 3-kinase (*PI3K*) results in reduced protein expression and constitutively increased activity of the PI3K pathway [4]. Similarly, the L1016S variant (rs61733127) in the PH domain and Leucine-rich repeat Protein Phosphatase 2 (*PHLPP2*) leads to reduced phosphatase activity and increased signaling through the AKT and Protein Kinase C (*PKC*) pathways (which are both substrates of PHLPP2) [5].

The article by Liu et al. adds to the growing list of publications that examine the functional impact of rare germline variants in the laboratory [6]. They studied a germline variant in the MET gene (Hepatocyte Growth Factor [HGF] Receptor), MET-T1010I (rs56391007), that occurs in <1% of the normal population but was observed in 2 % of metastatic breast cancer patients (n=240) in their study. It has also been previously linked to hereditary lung and colorectal cancer. They report that the expression of MET-T1010I in the MCF-10A immortalized breast epithelial cell line caused marked functional consequences including increased colony formation, cell migration, and invasion *in-vitro*. It also increased tumor growth *in-vivo* in human HGF transgenic mice. It is important to note that this SNP has not been associated with increased risk for developing breast cancer in large genome wide association studies and previous laboratory studies have reported conflicting functional effects for the T1010I variant in NIH3T3 fibroblast and murine myeloid BA/F3 cell line models as discussed by Liu et al.

These observations support the hypothesis that the effect of this SNP, and probably many others, is genomic context dependent. What constitutes a somatic cancer driver event may depend on the constellation of polymorphisms already present in an individual's germline, which could explain the lack of high frequency recurrent oncogenic mutations in breast cancer (other than PI3K and P53). This could also explain the cell line-restricted, transforming effects of many known oncogenes because several studies have shown that oncogenes do not transform every type of cell or cell line even from the same tissue. To further support the important role of inherited genetic variants in the biology of sporadic cancers, several recent studies have demonstrated that the prognosis of early stage breast cancer tends to cluster by families, suggesting a hereditary component that is independent of tumor characteristics and treatment [7]. Better understanding of the functional interactions between germline and somatic genetic events represents a fertile ground for research, and could fundamentally change the way we think about cancer biology.
